# DA_2DCHROM — a data alignment tool for applications on real GC × GC–TOF samples

**DOI:** 10.1007/s00216-023-04679-7

**Published:** 2023-04-10

**Authors:** Nikola Ladislavová, Petra Pojmanová, Štěpán Urban

**Affiliations:** Department of Analytical Chemistry, University of Chemistry and Technology Prague, Prague, Czech Republic

**Keywords:** Data alignment automation, GC × GC–TOF real samples, Data alignment algorithm, Human scent

## Abstract

Comprehensive two-dimensional gas chromatography coupled with mass spectrometry (GC × GC–MS) has great potential for analyses of complicated mixtures and sample matrices, due to its separation power and possible high resolution. The second component of the measurement results, the mass spectra, is reproducible. However, the reproducibility of two-dimensional chromatography is affected by many factors and makes the evaluation of long-term experiments or cross-laboratory collaborations complicated. This paper presents a new open-source data alignment tool to tackle the problem of retention time shifts — with 5 different algorithms implemented: BiPACE 2D, DISCO, MSort, PAM, and TNT-DA, along with Pearson’s correlation and dot product as optional methods for mass spectra comparison. The implemented data alignment algorithms and their variations were tested on real samples to demonstrate the functionality of the presented tool. The suitability of each implemented algorithm for significantly/non-significantly shifted data was discussed on the basis of the results obtained. For the evaluation of the “goodness” of the alignment, Kolmogorov–Smirnov test values were calculated, and comparison graphs were generated. The DA_2DChrom is available online with its documentation, fully open-sourced, and the user can use the tool without the need of uploading their data to external third-party servers.

**Graphical Abstract:**

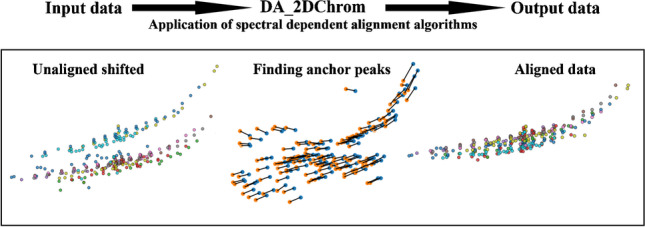

**Supplementary Information:**

The online version contains supplementary material available at 10.1007/s00216-023-04679-7.

## Introduction

Two-dimensional comprehensive gas chromatography coupled with mass spectrometry (GC × GC–MS) [1] is a powerful technique combining the high-resolution potential of two-dimensional separation and the identification ability of mass spectrometry techniques (such as time-of-flight and quadrupole). Exploiting the reproducibility of mass spectra obtained under the same conditions is a well-established approach, and there are many commercial mass libraries. On the other hand, evaluating chromatograms measured under the same conditions is biased by various factors, such as column ageing, column flow fluctuations, or change in column length caused by their replacement. As a result, retention time shift complicate data alignment and pairing of peaks of the same chemical compounds in different chromatograms. Thus, data alignment is a crucial step, and its correct execution eliminates downstream errors for further evaluation of the results.

To demonstrate such situation when these obstacles come in play, we have used our research of human scent as an example. The data collection spreads over a 2-year span. During this time, various maintenances occurred causing different time shifts. Since the research is focused on non-target analysis, a special library of identified chemical compound had been developed. This library is based on one of the algorithms discussed later in this paper (MSort) and, ideally, should keep special codenames for each detected chemical compound. Without proper data alignment, the risk that compounds in the measured chromatograms may be labelled with the wrong codename (or one compound could have many such codenames) is significantly higher. These errors are hard to detect in later stages of data processing such as statistical analysis — thus, they are propagated into the later stages of data processing.

There are commercial software programmes available to help overcome the problem of retention time shifts; the time correction tool is sometimes part of the official software supplied by the instrumentation manufacturer. However, these programmes are often patented, or their core code is protected by other means, making such software one of the “black box” steps involved in data treatment. This paper introduces an open-source tool to alter these commercial options and aims to contribute to the open-science effort in a field of GC × GC–MS data processing automation.

Data alignment algorithms discussed in this paper can be divided into two groups: those with spectral dependency and those without spectral dependency. Algorithms without spectral dependency are suitable for GC × GC-FID (two-dimensional gas chromatography coupled with flame ionization detector) application because they work only with the peak coordinates (respectively, they fit two signals based on their shape and magnitude). Coherent point drift (CPD) [2, 3] fits one point set onto the other by maximizing the likelihood of both point sets. This approach treats data alignment problem as a probability density estimation problem and alignment itself is performed by applying a transformation function on the data. The transformation function has three parameters for rigid transformation — rotation, translation, and scaling. Non-rigid transformation then allows non-uniform scaling and skewing of the data during the transformation. Li et al. [4] developed a spectral similarity extension algorithm for CPD, which should outperform local alignment algorithms in cases of real data with a high density of peaks. One of the most favourite data alignment algorithms is correlation optimized warping algorithm [5, 6]. This algorithm works on the raw data level by splitting the chromatogram into subregions. Each subregion is then fitted to the “template” chromatogram. This algorithm is ideal for samples which do not vary in their qualitative composition.

There are semi-automatic alignment algorithms without spectral dependencies [7, 8] that require the user to manually enter the list of anchor points (peaks present in both referential and aligned chromatograms) for each aligned chromatogram. The requirement of manual intervention is an undesirable characteristic with respect to the current emphasis on automatization. However, the concept of anchor points — points present in all aligned samples, is a pivotal idea for spectral-dependent algorithms. Gros et al. [7] used the user-predefined list of anchor points to align data through a linear extrapolation in the first dimension and Sibson natural neighbour interpolation in the second dimension. Reichenbach et al. [8] used the concept of anchor peaks to create the transformation grids for data alignment. A modified anchor peak algorithm (RI) had been used in our laboratory. There were 17 anchor points in total. Thirteen anchor points were used for alignment of data in the first dimension, and 1 shared plus 4 unique anchor points were used for alignment in the second dimension. Both retention times were separately recalculated to retention Kovats indices, and this step aligned the data without any other data manipulation.

**Fig. 1:**
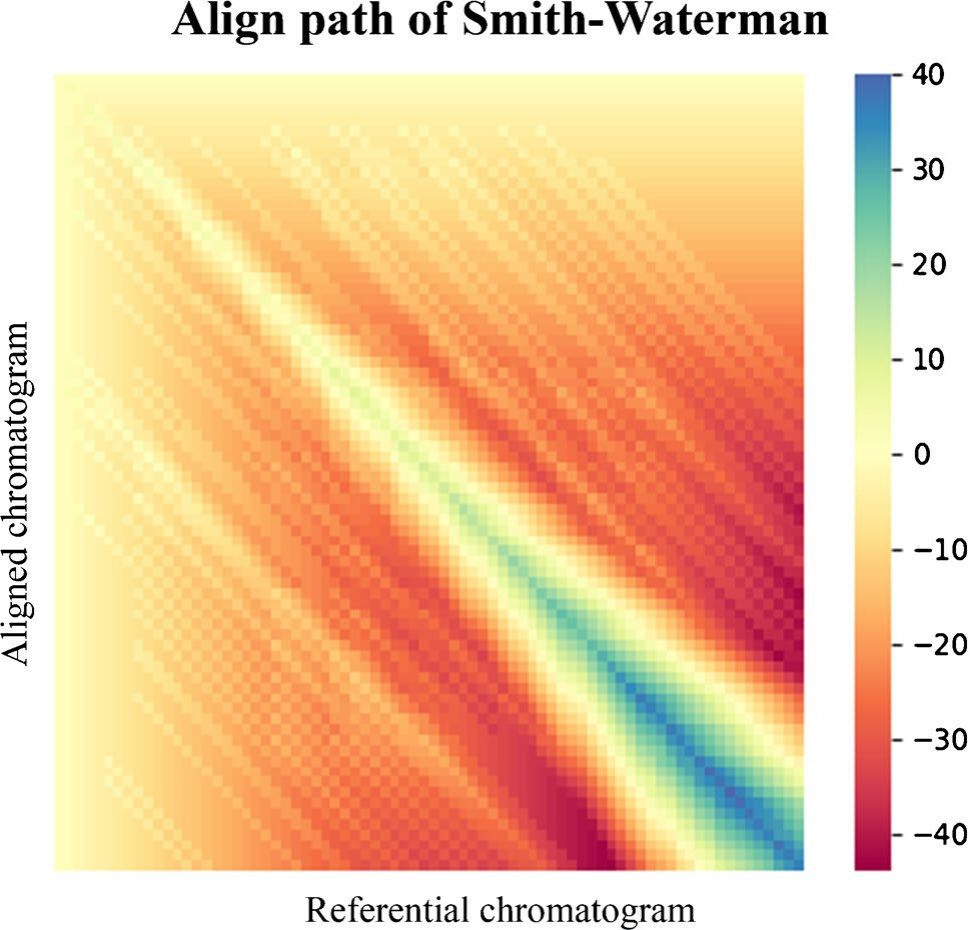
Visualization of the Smith–Waterman algorithm. The ideal aligning path follows the highest scoring pixels back the cell near [0,0] position — the first cell in the matrix. This is a common algorithm behaviour for similarly populated chromatograms. However, if the number of peaks in the aligned chromatograms differs significantly, the aligning path often initiates in a cell further away from the origin [0,0]. Each pixel represents one cell of the matrix

Spectral-dependent algorithms include not only peak coordinates but also spectral similarity between peaks. The Smith–Waterman algorithm [9] originally used in proteomics [10] was modified with respect to spectral similarity. The “match”/ “mismatch” decision rule depends on whether the spectral similarity exceeds the given threshold. Each cell of the aligning matrix (*n* × *m*, *n* dimension corresponds to the number of peaks in the aligned chromatogram, the *m* dimension corresponds to referential chromatogram) is then scored as a sum of the “match”/ “mismatch”/ “gap” rule and the value of the previous cell. Then, it traces back, from the end cell with the highest score, the path with the highest scoring values, which is the resulting “aligning” path (see Fig. 1).

Robinson et al. [11] altered the decision rule. Each cell of a scoring matrix is calculated from the similarities of the peak powered to the time difference between the reference and the aligned peak (the time difference is calculated from the total retention time). BiPACE 2D [12] builds on the previous dynamic programming methods. BiPACE 2D treats the 1st and 2nd retention time shifts separately, thus adding one more parameter to the Robinson equation [11, 12]:

1 $${f}_{2d}\left(p,q\right)=\mathrm{exp}\left(-\frac{({t}_{1,p}- {t}_{1,q}{)}^{2}}{2{D}_{1}^{2}}\right)\bullet \mathrm{exp}\left(-\frac{({t}_{2,p}- {t}_{2,q}{)}^{2}}{2{D}_{2}^{2}}\right) \bullet s(p,q)$$

*s(p, q)* is the spectral similarity of the compared peaks; the exponential members of the equation take the squared difference between the retention times for the first and second dimension divided by tolerance parameters *D.* The higher the *D* value, the greater the time differences between the peaks tolerated. The exponential terms are called retention time (RT) penalty terms, and the user can assign an additional threshold parameters *T*_1_ and *T*_2_ and thus apply a search window discrimination to the peak comparison. The algorithm creates *Best Bidirectional Hits* tables (or maps) where the strength of the anchor point (peak, respectively) is expressed as a count of connections to the other members of the specific anchor point cluster (peaks of the same chemical compound through all aligned chromatograms). DISCO [13] identifies anchor points based on their Euclidian distances. First, the retention times are recalculated to the *z scores* (Fisher transformation). Then, *X* nearest peaks (20% of the total number of all peaks in the table was set as a discrimination rule for this experiment) from the referential chromatograms are compared to the inspected peak from the aligned chromatogram. If the spectral comparison exceeds the critical threshold, the inspected peak is marked as the anchor point. The overall execution of the alignment starts with a randomly picked chromatogram as the referential table of the anchor peaks. Through the iteration over all aligned chromatograms, anchor peaks which are not found in all samples are dropped, and the final referential table is a list of peaks found in every member of the aligned dataset. The alignment itself is done by a linear fitting method. MSort [14] has a static search window for the first and second retention times and chooses a peak with the highest spectrum similarity coefficient in the searched area. PAM [15] has no search window. Like DISCO, PAM recalculates times to Canberra distances, and the selection rule is a weighted product of the distance and spectral similarity of peaks. The algorithm compares all peaks from both chromatograms, and matches the peaks with the highest similarity according to [15]

2 $${M}_{d}\left({t}_{j},{r}_{i}|w\right)=w\bullet (1+{D}_{d}\left({t}_{j},{r}_{i}\right){)}^{-1}+(1-w)\bullet S\left({t}_{j},{r}_{i}\right)$$

where *w* is weight parameter, *D*_*d*_ is the distance between the two compared peaks (*t*_*j*_, *r*_*i*_) and *S* is the spectral similarity of the two compared peaks. To speed up the calculation process, we added a similarity threshold parameter. If the spectral similarity of the two compared peaks does not exceed the threshold, the rest of the calculation is skipped. Based on the obtained results, the new variation of previously discussed algorithms was proposed — the non-targeted data alignment (TNT-DA) — see the Experimental section.

As all tested algorithms (BiPACE 2D, DISCO, Msort, PAM, and TNT-DA) were of the spectral dependant family, the final DA-2D_Chrom tool [16] provides optional parameters for the mass spectra comparison. Kim et al. [17] tested various approaches to mass spectra comparison and the influence of mass spectra transformation. Four mass spectra comparison setups published by Kim were tested in this study: Pearson’s correlation and dot product, both with and without spectra transformation.

The main motivation of this experiment was to develop open-source alignment tool with multiple algorithms implemented in ready-to-deploy state. The tool was then used for the peak alignment of real human scent samples acquired by a non-target analysis approach and evaluated the potential of algorithms previously described in various studies. In the following paragraphs, all implemented data alignment and spectral comparison methods are tested and compared using DA_2DChrom. First, all implemented algorithms are used for data alignment of small sample set [18]. The data alignment problems (mass spectra deformation after deconvolution, misidentification of anchor points) encountered during the DA_2DChrom development and their impact on data alignment are also briefly discussed. In the second part of data alignment experiments, the best performing published algorithm and TNT-DA are applied to the complete dataset of 503 scent samples [19]. The ability to perform a good fit peak alignment on a large set of samples acquired over a long period of time or even by different laboratories (following the same methodology) would greatly improve the potential of data sharing in a GC × GC community.

## Experimental

### Sample data set

All human scent samples were acquired with the same methodology and measured under the same settings. Both steps were described in our previous work [20], a detailed description is provided in Supplementary materials: S1_Methodology. The 503-chromatogram dataset could be divided into three subgroups: system_1 (274 samples), system_2 (136 samples), and system_3 (93 samples). A maintenance operation (column replacement in both dimensions) was performed between the subsets of the measurements. The main idea of the split is that there should be minimal time shift variation between chromatograms from the same subset (homogenous samples) and a larger time shift variation when comparing samples from different subsets. All samples were pre-processed with ChromaTOF® software (version 4.72.0.0, LECO Corp., St. Joseph, MI, USA) at signal-to-noise (S/N) ratio levels 100, 300, and 500. Thresholds below S/N 100 detected many “noise” peaks with incomplete mass spectra which would be excluded from real analyses anyway. The detected peaks (1st dimension retention time, 2nd dimension retention time and spectra) were exported without any additional adjustments of the data to a.csv file format with a tabulator as a separator. To test the “goodness” of the data alignment for the each implemented algorithm, training data set containing twenty samples were randomly chosen as a training set [18] (8 from system_1, 3 from system_2, and 9 from system_3). Those samples were additionally re-exported with columns bleeding areas excluded. For testing the performance of Pearson’s correlation versus cosine similarity as a tool for mass spectra comparison, five standard samples of 48 compounds were measured under the same conditions as samples from system_3 (see Supplementary materials: S3_Table of standards).

### Volunteers and naming of the samples

In total, samples from 40 volunteers (20 women and 20 males) through all three system conditions were processed. The samples are named in a uniform way — first letter represents a biological sex of the volunteer, the following number is the ID of the volunteer in a database, and after the underscore, the ID of the sampling session follows. (M15_8 means that this volunteer is the 15th male volunteer in our database, and this sample is their 8th sample provided.)

### Tested algorithms

BiPACE 2D, DISCO, MSort, PAM, Smith–Waterman, TNT-DA, and additionally the modified anchor point algorithm were tested with multiple parameters if possible. In some cases, algorithms were modified (see following sections). Note that the modified anchor point algorithm was not tested on all 503 chromatograms, as finding the best automated solution was the main objective of this study.

To minimize the differences between algorithm executions, the new data alignment tool DA_2DCHROM [16] was designed in a manner to execute the alignment always in the same manner, so the execution differed only in the selection rules based on the chosen algorithm (step *Find anchor peaks*). Sample system_1_M13_4 was set as a referential chromatogram and the rest of the 19 samples were aligned, one by one, to the referential chromatogram. Each alignment process executed the following steps:

*Find anchor peaks → Check elution order → Export anchor points → Retention time shift correction → Export peak map → Export time corrected chromatogram.*

The selection rules and tuneable parameters for the alignment tool (step *Find anchor peaks*) are summed up in Table 1.

**Table 1:** Summarization of the inspected algorithms and their parameters used in DA_2DCHROM [16]

Data alignment algorithm	Search window	Selection rule	Tuneable parameters
BiPACE 2D	Optional (*T* parameters)	Equation 1	*D*_*1*_*, D2, T*_*1*_*, T2, spectral similarity*
DISCO	Optional (20% set as default)	*Top spectral similarity*	*Spectral similarity*
MSort	Yes	*Top spectral similarity*	max_shift_1 [s], max_shift_2 [s], spectral similarity
PAM	No	Equation 2	*w, spectral similarity*
Anchor peaks (RI)	No	None	None
Smith–Waterman	No	Spectral similarity	Spectral similarity
TNT-DA	Optional (20% set as default)	Equation 2	Distance method, *w*, spectral similarity

### The non-target data alignment tool (TNT-DA)

TNT-DA is derived from the DISCO discrimination rule and the PAM selection rule. First, the retention times are recalculated to *z *scores. For every peak in a referential table, *Canberra* or *Euclidian* distances for every peak in an aligned table are calculated. Then, 20% of the nearest peaks are considered as potential anchor peaks. Spectral similarity is calculated for every potential anchor peak. If the spectral similarity reaches the similarity threshold, the PAM selection rule is applied, and the peak from the aligned table with the highest score is marked as an anchor peak. The performance of the proposed algorithm was tested, and the best result was compared to the other algorithms.

### Performance evaluation

First, the distributions of the peaks in the chosen 2D chromatograms were compared for each dimension using a Kolmogorov–Smirnov (K-S) test [14, 21]. It is a nonparametric test of the proximity of two distribution functions. The closer the value to the zero, the better the fit of the distributions. That means that unaligned data would have higher K-S value than perfectly aligned data (the zero value could never be reached with our dataset as the samples were not qualitatively identical). The overall processing times of the test samples were inspected. The K-S test values were then recalculated after the peak alignment of the inspected dataset, and the pre-alignment and after-alignment values were compared. The F1 score was used to compare Pearson’s correlation versus cosine similarity as a tool for comparing distances between mass spectra. F1 score is the weighted average of precision and recall [22]:

3 $${F}_{1}=\frac{TP}{TP+\frac{FN\;+\;FP}{2}}$$

where *TP* = *true positive*, *FP* = *false positive*, and *FN* = *false negative*.

All results (graphical and numerical) discussed in the section “Results and discussion” are part of the provided sample dataset [18]. Exported *m/z* values are representing relative abundance of the *m/z* fragment in the obtained spectrum. Please, see the dataset Readme file. All calculations were performed on a common workstation (Intel® CoreTM i7-9700F @ 3.00 GHz, with 32 GB RAM DDR4 and 64-bit Win 10 OS).

## Results and discussion

The following sections demonstrate the impact of switching between alignment algorithms and tuning of their parameters while using the introduced open-source data alignment tool. As the development of the 2D_Chrom involved lot of testing and visualizations of failed experiments, the main pitfall of automated data alignment algorithms was inspected — misidentification of the anchor peaks. The symptoms and solutions of this phenomenon are discussed in following paragraphs.

### Time efficiency

The least demanding algorithm was a modified anchor point (RI). The execution of data alignment of one sample took less than 1 s. However, it took approximately 15–20 min to identify, mark, and export anchor peaks. Algorithms with a “search” window (MSort, DISCO, BiPACE 2D, TNT-DA) took the same amount of time (15–20 min) for one sample. The PAM algorithm was slightly slower, and the execution of one sample took around 40 min to 1 h. The reason of lower time efficiency is the fact that PAM algorithm has no search window, or any other discrimination step; thus all peaks are compared against each other. The execution of the Smith–Waterman algorithm was counted in hours and exhibited severe misalignments while processing chromatograms with fewer peak counts, thus being left out of further testing.

### Sample dataset alignment

**Fig. 2:**
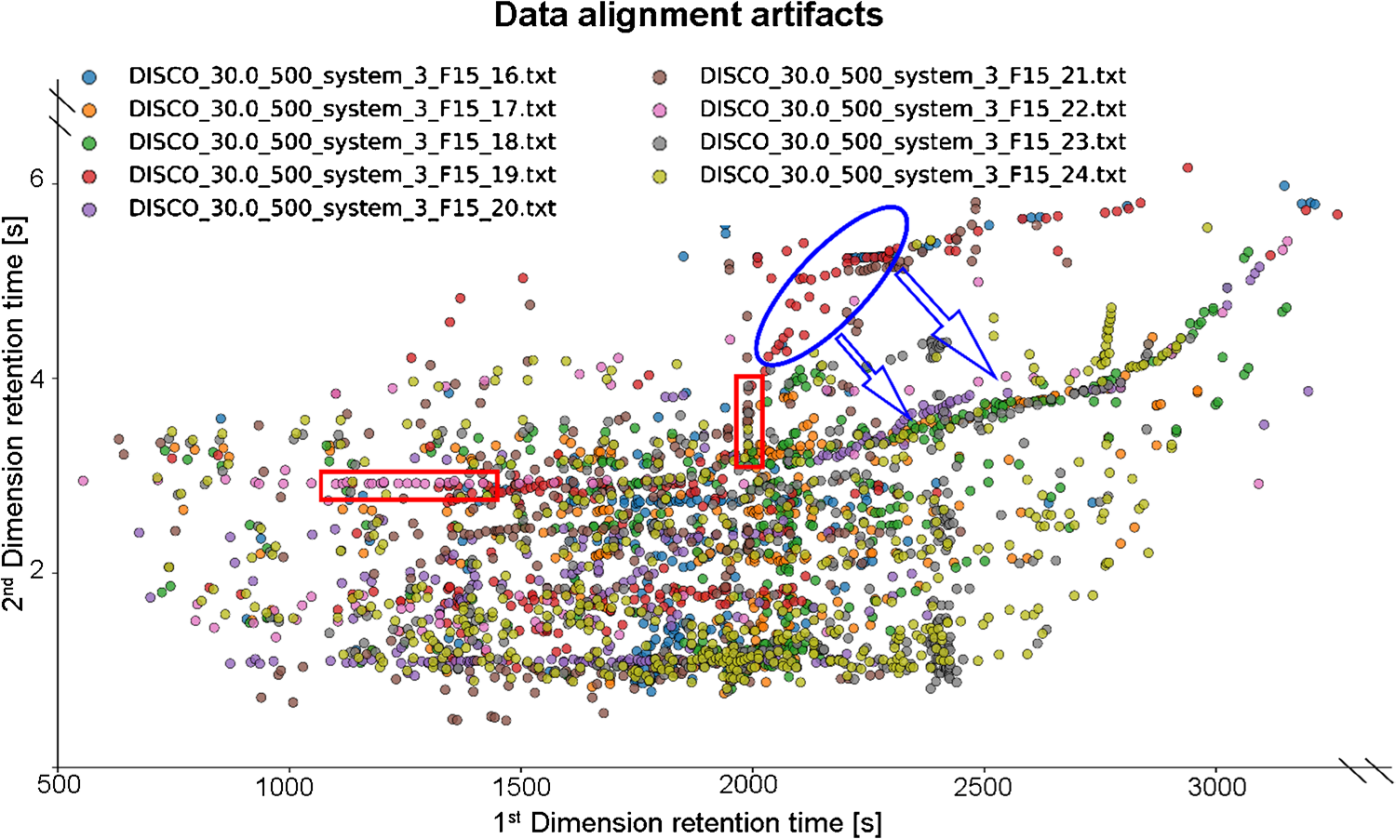
An example of alignment artefacts in one of the aligned samples. Each colour represents a different chromatogram. Red boxes represent the wrong alignment in the first and second dimensions. The vertical red box (second dimension misalignment) clearly causes alignment error for samples F15_19 and F15_21 in the 1st RT > 2000. The displacement is market by the blue ellipse, and the expected direction of the alignment is pointed out by the blue arrows. These results were obtained using DISCO algorithm, S/N = 500, and spectral similarity threshold (Pearson’s correlation coefficient) was set to 0.3

**Fig. 3:**
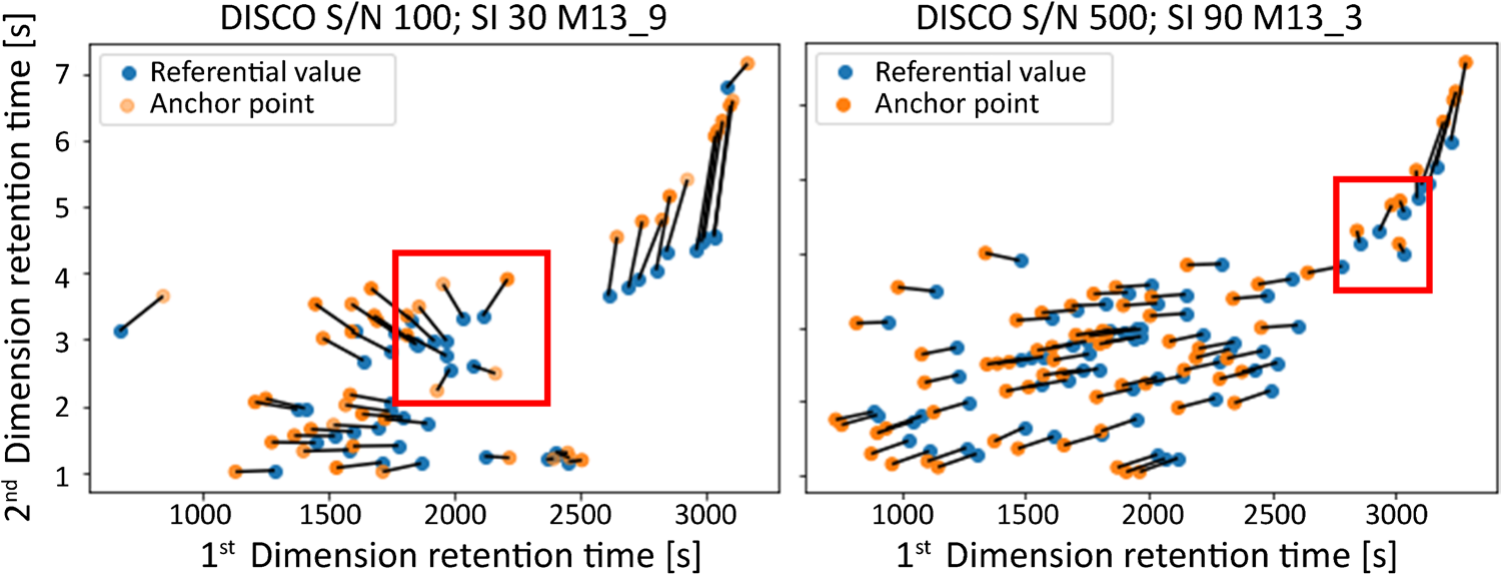
An example of anchor peak misidentification. On the left (DISCO algorithm with spectral similarity threshold ≥ 30%), obvious identification errors are marked with red boxes. On the right (DISCO algorithm with spectral similarity threshold ≥ 90%), there is an example of better anchor peak identification. However, even this experimental alignment exhibited identification errors for the area *x* > 2700 s (elution time greater than 2700 s)

For DISCO, MSort, BiPACE 2D, and PAM, “alignment artefacts” appeared. They can be described as horizontal or vertical lines formed by misplaced aligned points (Fig. 2). Homologous series tend to have linear or exponential character in a later stage of the analysis (corresponds to the isothermal temperature end of the chromatographic programe) in the proposed experimental setup. Therefore, the rearrangement of the detected peaks to constant function behaviour marks an error in the data alignment. The appearance of alignment artefacts was correlated with the incorrect identification of the anchor peak — the reference table peak which was identified in an aligned table. This misidentification was a result of either low critical value of spectral similarity or wrong setting of the discrimination rules (for example, favouring distance between two peaks over spectral similarity). Figure 3 depicts such an incorrect identification. Time shifts in both dimensions have similar direction through the sample; therefore, different directions of the shift are an indicator of the identification error, which eventually results in poor alignment of the chromatograms.

The evaluation of the K-S metrics was misleading in some cases. For example, the dataset contained dense data, and the overall distribution of peaks differed in the first place (some of the samples had richer polar fractions than others). Second, a denser area along the alignment artefact lines distorted the resulting distributions. Considering the previous points, all alignment experiments were additionally visually checked for the performance evaluation.

BiPACE 2D was tested with a different parameter configuration. Cut-off values *T*_1_ and *T*_2_ were both set to value 0.99. *D*_1_ (threshold for time delta in the first dimension) was tested at levels 10, 50, and 100 in combination with *D*_2_ (threshold for time delta in the second dimension) values 0.25, 0.5, and 0.8. Performance improved with increasing S/N (100 <  < 300 < 500) and the *D*_1_ parameter. Even with the alignment of the highest *D*_2_ value (0.8), the alignment of the data in the second dimension was not sufficient, especially with second dimension retention times *y* > 4 s. For DISCO, the performance improved with increasing spectral similarity (30% <  < 80% < 90%) and with increasing S/N. MSort proved to be sensitive to user input (search window boundaries). The correctness of the alignment was raised with broader boundaries (especially for the second dimension) and a higher threshold of similarity of the mass spectra. However, the user must set the boundaries with respect to distances between spectrally similar elution areas (in second dimension) or members of homologous series (in first dimension) to avoid anchoring on the wrong peaks. The PAM algorithm is rather time-consuming but, on the other hand, less discriminating than other inspected algorithms. Its sensitivity to various time shifts could be managed through the preference of spectral similarity (*w* parameter is a weight parameter — higher values prioritize distance between the peaks, lower values prioritize the spectral similarity) without any processing time trade-off. The value of *w* = 0.8 yielded the best results when considering the alignment in both dimensions.

All algorithms had one thing in common, their performance improved with a rising S/N. This could be caused by improper peak deconvolution done by software used for peak detection and data export. For fully separated peaks, the level of S/N does not impact the correctness of the presented mass spectra. Compared to that, the coelution of peaks caused disjunction of spectra because of wrong deconvolution. The most common example in the presented samples are higher ethyl esters of fatty acids and their saturated and non-saturated pairs, which were coeluting heavily. With lower S/N ratios, there were more than 4 peaks detected instead of two. The real peaks were often split into two “peaks”, one with spectrum containing lower *m/z* fragments and the other with characteristic masses (236 etc.) as base fragments (fragments with the highest intensity). As all the tested algorithms rely on the mass spectra reproducibility, the variations in spectra lead to the processing errors (such as spectral similarity of the peaks of the same chemical compound do not exceed the similarity threshold through the samples or the algorithm identifies the wrong member of the homologous series).

For the complete commentary on the performance of all implemented algorithms, every algorithm and its parameter tuning were, in fact, tested twice. The final DA_2DChrom tool contains step *Check elution order*. When this step was left out of the alignment procedure, the number of anchor peak identification errors (mainly for mass similarity thresholds 30 and 80%) was dramatically higher (most of them were false identifications) compared to the situation with this step involved. For example, for mass similarity > 30%, there were hundreds of detected anchor peaks for each chromatogram. However, with the *check* step on, the count of the anchor peaks seldomly reached over 150. At the early stages of the DA_2DChrom, the *elution check* was performed separately for the 1st dimension and the 2nd dimension. This approach, however, had issues while dealing with the peaks with the same retention times in one of the dimensions. Thus, the implemented *elution check* checks on the elution order with respect to the total elution time (sum of the retention times from both dimensions).

In terms of data alignment, the modified anchor point algorithm (based on retention indices) outperformed other algorithms. There are two possible outcomes of this finding: first, when the user is dealing with a reasonable count of complicated samples, human processing time should be an acceptable trade-off for the algorithm performance. Second, if the user is familiar with the structure and common pattern in aligned chromatographs, there is a possibility to write a list of selection rules (considering spectral similarity and rigid relative distances) for finding such anchor peaks automatically, which might be a very challenging task. For the completion of the study, the modified anchor point algorithm was used as a pre-process alignment, and all other algorithms were applied on the transformed dataset.

**Fig. 4:**
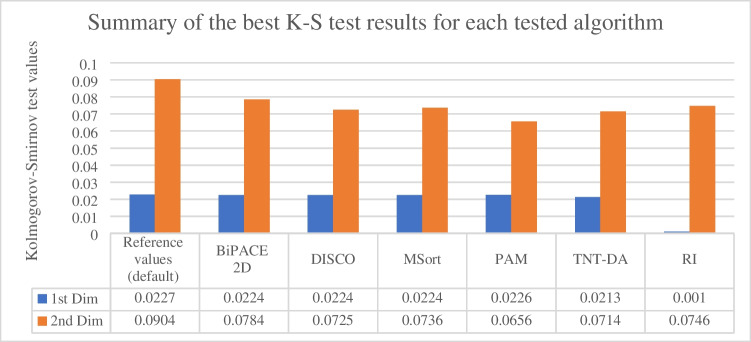
Summary of the best K-S test results obtained for each algorithm (with paramaters). Blue bars represent values for the 1st dimension, orange bars represent results for the 2nd dimension. BiPACE 2D settings: *D*_1_ = 100, *D*_2_ = 0.8; DISCO settings: Pearson’s correlation = 0.8; MSort settings: Pearson’s correlation = 0.8, search window =  ± 25 s in the 1st dimension, ± 0.5 s in the 2nd dimension; PAM settings: w = 0.8; TNT-DA settings: cosine correlation = 0.9, w = 0.4, distance = Canberra

Figure 4 sums up K-S test results for all tested algorithms and their top performing parameters. For full reference, see Supplementary materials: S2_KS-test_results_SN_500. Graphical results are part of the published dataset [18], which was created as a part of this study. The RI approach was the best performing in the 1st dimension, while the PAM algorithm reached the best result in 2nd dimension. As the final dataset contained 503 samples, we decided to prioritize DISCO over PAM and RI because of the time efficiency in the case of PAM and fully automated concept in the case of RI.

#### Modified anchor point algorithm as a pre-processing step

The pre-align step led to higher processing times of all algorithms (except PAM). Surprisingly, the performance of BiPACE 2D and DISCO was worse than without the pre-align step (meaning that there were more align artefacts and the K-S test results achieved higher values in both dimension). MSort performed better as time shifts were reduced. The PAM results were not different from the alignment based on the raw data. Overall, considering the input effort, using modified anchor point algorithm as a pre-processing step has no positive effect on the quality of the final data alignment.

#### Spectral transformation and spectra similarity comparison

As spectral comparison was the main issue, additional options of spectral similarity were inspected [17] — spectral transformation and cosine similarity [23]. The best performing transformation step, according to Kim’s paper, was the following: the intensity of each mass value was powered to 0.53, and the mass value was powered to 1.3 — this approach favours the masses with higher *m/z* ratios, which is, for example, useful while distinguishing between members of homologous series.

For this sub-experiment, five chromatograms of the standard mixture (48 compounds) were measured, the peaks were manually identified, and subsequently, only the mass spectra were compared. The first chromatogram was set as a reference chromatogram. *F*_1_ score [22] was used as an evaluation metric. The Pearson’s correlation coefficient without transformation reached the *F*_1_ score 0.61 (precision = 0.44, recall = 0.97), and the spectral transformation with coefficients 0.53 and 1.3 improved the *F*_1_ score to 0.87 (precision = 0.89, recall = 0.86) (see Table 2). Cosine similarity with the same spectral transformation scored 0.90 (precision = 0.83, recall = 0.98). However, transformed Pearson performed better from the precision perspective. The higher occurrence of *false* positive results in the case of cosine similarity is acceptable when the step of *Check elution order* is employed in the script. These results were applied to the development of the data alignment software, and all available algorithms were supplied with cosine similarity as an option for mass spectra comparison.

**Table 2:**
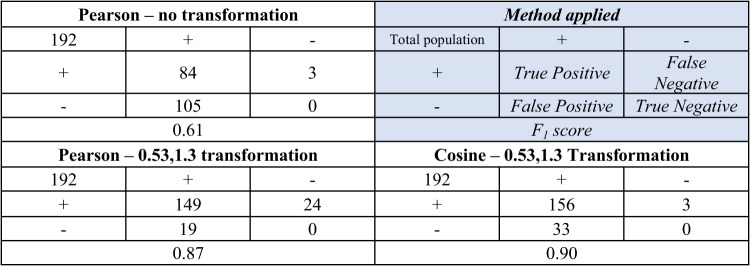
Confusion matrix of the spectral similarity comparison methods. True positive = correct identification; false positive = compound from the reference table had a higher spectral correlation with the other compound from the compared table (wrong identification); false negative = none of the reference table — compound pairs from align table had not reached the threshold of 80%; true negative was 0 since all compounds were present in the 5 chromatograms)

### The non-target data alignment tool (TNT-DA)

**Fig. 5:**
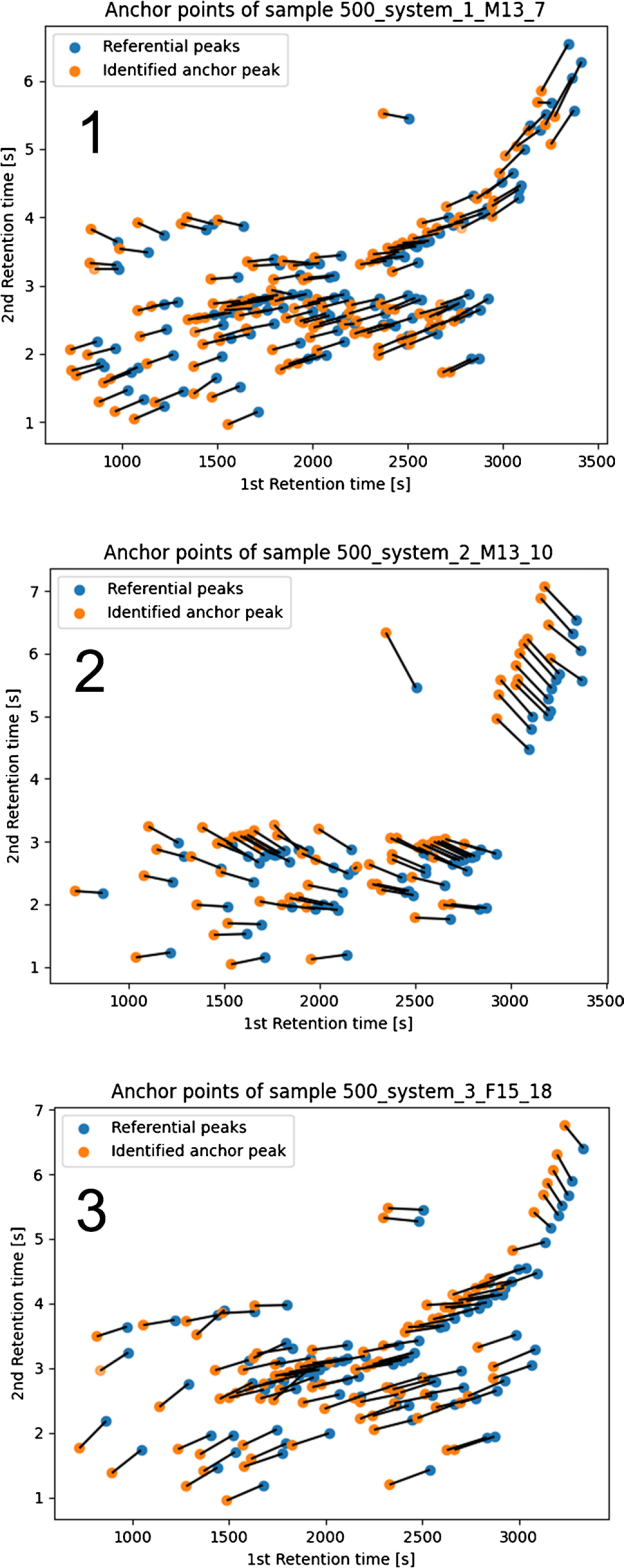
Map of the identified anchor peaks (TNT-DA) and their shift against referential peaks. The top (1) map represents the shift for a sample from the very same system (system_1), the middle (2) map shows the shift for a sample from system_2, and the bottom (3) map depicts shift for a sample from system_3

**Fig. 6:**
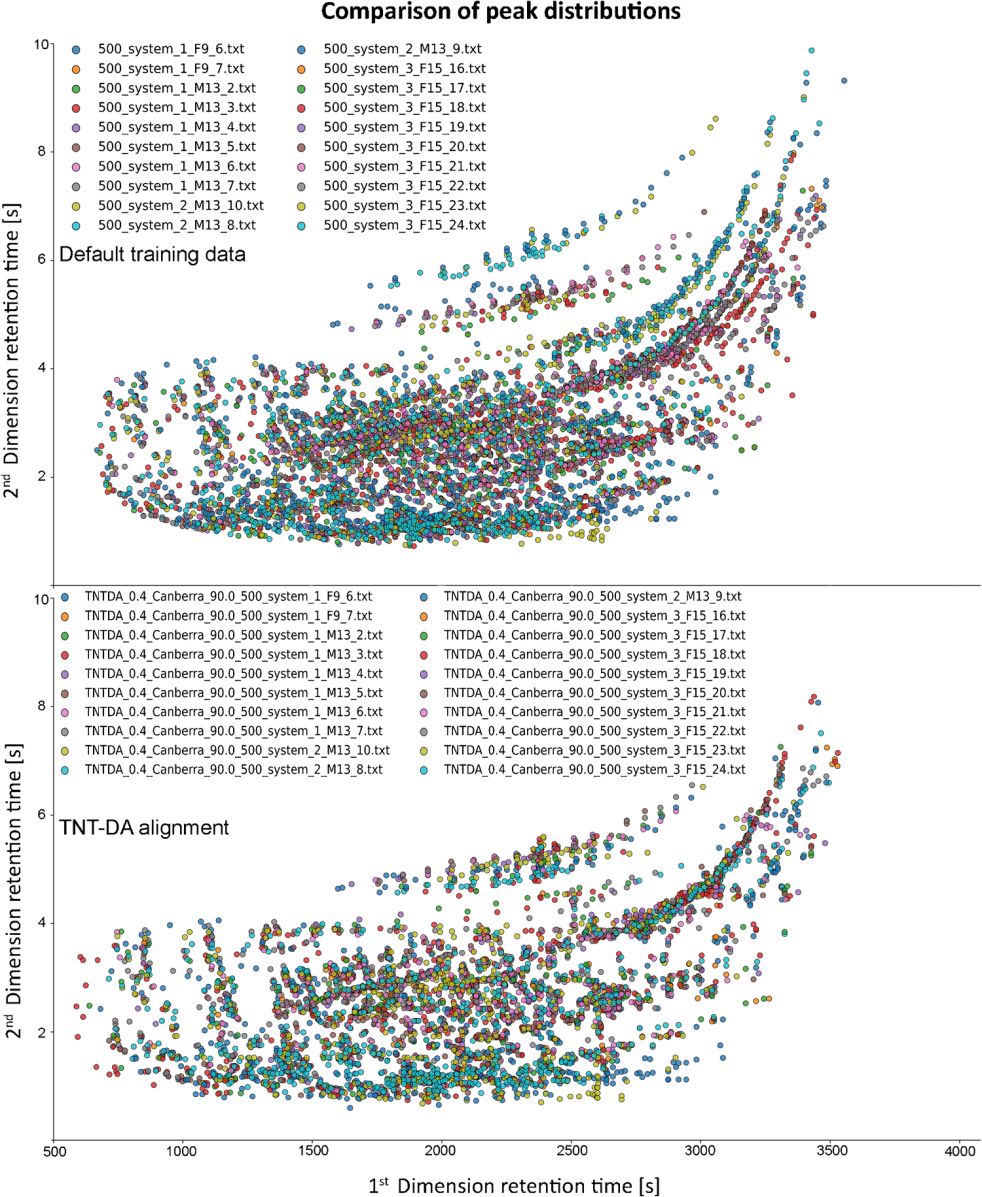
Visual comparison of default training data (top) and data aligned with TNT-DA (bottom). Both data sets were exported on S/N 500. TNT-DA was performed with w = 0.4, distance method = Canberra, similarity threshold = 90%. The spectral data were transformed (0.53, 1.3) and compared by cosine similarity

The best performing setup included *Canberra* distances, cosine similarity (0.53, 1.3 transformation) with threshold of 90 % and the *w* =0.4 (Fig. 4). The K-S test value for the 1st dimension was 0.0221 (default value = 0.0227) and 0.0714 for the 2nd dimension (default value = 0.0904). Figure 5 demonstrates the anchor peak shift for one sample from each system. The overall alignment of the data is shown at Fig. 6.

### Overall data alignment

For the final data alignment experiment, the best performing tested algorithm (DISCO as the compromise between time efficiency and goodness of the alignment — also this algorithm is most similar to TNT-DA) and TNT-DA were tested on the complete dataset containing 503 samples. On the basis of the results of the sample dataset alignment, DISCO selection rules and cosine similarity with spectral transformation were chosen as the best alternative considering the processing time demands and the achieved alignment of the data points. The spectral similarity threshold was set at 90 % (0.9) for both algorithms. The TNT-DA *w* parameter was set to 0.4, and the Canberra distance was applied for the calculations. The K-S result (default values were 0.0283 for the 1st dimension and 0.1138 for the 2nd dimension) for the TNT-DA was 0.0282 for the 1st dimension and 0.0819 for the 2nd dimension, and DISCO scored 0.0280 for the 1st dimension and 0.0812 for the 2nd dimension. The processing time was roughly the same in the dozens of minutes per sample. For visual comparison, 100 randomly chosen pairs were exported as distribution maps and visually compared (note that random.seed was set to 42). For both algorithms, alignment artefacts appeared (see published Training dataset [18]) and the K-S results were similar. The results suggest that DISCO might be more error prone when dealing with dense and unadjusted data (the small testing sample set used for primary testing and on which TNT-DA performed better was re-exported without column bleed regions). The TNT-DA algorithm does not dramatically improve the data alignment compared to the tested algorithms but provides additional tuneable parameters if the user needs more flexible tuning.

## Conclusions

In total, more than 1100 data alignment steps were carried out during this study using newly introduced open-source data alignment tool — DA_2DChrom. The performance differences of each implemented data alignment algorithm were demonstrated. For every algorithm with tuneable parameters, multiple alignment runs were carried out and compared to each other.

First, orientation experiments were performed on a reduced sample set containing twenty samples with the following result: BiPACE 2D has four controllable parameters. For our study, the discrimination rules *T*_1_ and *T*_2_ were rigid and only *D*_1_ and *D*_2_ were varied during the experiments. Adjustable parameters allow BiPACE 2D to deal with various time changes, but the algorithm must first be tuned. The DISCO algorithm is robust considering time shifts, and swift has no real tuneable attributes, which makes DICSO a very user-friendly approach. MSort is perfect for chromatograms with smaller time deviations. It has the quickest execution time, and if there is no significant time shift, MSort would be the best choice. The PAM algorithm is the most time-consuming. However, it does not discriminate any peaks in the comparison itself. The user can choose the weight of the spectral similarity and peak distance on a single parameter. The PAM should be error prone to significant time shift as well as to different peak distribution. The processing time of the PAM algorithm could be reduced by introducing the “search window” boundaries (like MSort or BiPACE2D). The transformation of retention time coordinates into retention index coordinates proved to be very efficient, especially in the 1st dimension, but this approach is not viable in automation of data processing. Smith–Waterman algorithm was excluded from further calculations due to time-consuming calculations and severe misalignments for unequally populated chromatograms.

Choosing the right mass spectrum comparison method proved to be crucial and significantly improved the effectiveness of the algorithms. Thus, the DA_2DChrom was expanded for another tuneable argument for each algorithm — the user can choose between the Pearson’s correlation or cosine similarity when comparing mass spectra. The Pearson’s correlation reached higher *precision* score than cosine similarity, which, on the other hand, yielded better recall results. The user can also transform the mass spectra in order to favour characteristic masses (masses with higher *m/z* ratios).

In the second part, the TNT-DA algorithm, combining the speed of DISCO and selection rule of PAM, was proposed. The abilities of TNT-DA were compared to that of DISCO. Both algorithms were applied to the complete dataset (503 samples) and reached similar scores. TNT-DA is also implemented in the DA_2DChrom tool.

The performance of the DA_2DChrom can be improved by filtering out the peaks detected in the elution areas of column bleeds of both dimensions (this applies to the implemented algorithms in general). Subsequently, the results should be checked and realigned by different algorithm setups if needed.

The proposed algorithm TNT-DA and more importantly the alignment tool can be further developed and updated as the main goal of this work is to help overcome data alignment problem for users relying on commercial processing software often supplied by instrument manufactures. With sufficient data alignment, non-targeted analyses of two-dimensional chromatographic data could be as close to the routine as they are for one dimensional GC methods. The script used for the experiments as well as its documentation and the dataset are fully available online.

## Supplementary Information

Below is the link to the electronic supplementary material.

Supplementary file1 (PDF 95 KB)

Supplementary file2 (XLSX 34 KB)

Supplementary file3 (PDF 16 KB)

## Declarations

### Source of biological material

The biological material (skin smear and sweat) was taken from human volunteers.

## References

[1] Liu Z, Phillips JB (1991). Comprehensive two-dimensional gas chromatography using an on-column thermal modulator interface. J Chromatogr Sci..

[2] Myronenko A, Song X (2010). Point set registration: coherent point drift. IEEE T Pattern Anal..

[3] Deng B, Kim S, Li H, Heath E, Zhang X (2016). Global peak alignment for comprehensive two-dimensional gas chromatography mass spectrometry using point matching algorithms. J Bioinf Comput Biol..

[4] Li Z, Kim S, Zhong S, Zhong Z, Kato I, Zhang X (2020). Coherent point drift peak alignment algorithms using distance and similarity measures for two-dimensional gas chromatography mass spectrometry data. J Chemometr..

[5] Zhang D, Huang X, Regnier FE, Zhang M (2008). Two-dimensional correlation optimized warping algorithm for aligning GC×GC−MS data. Anal Chem..

[6] Tomasi G, van den Berg F, Andersson C (2004). Correlation optimized warping and dynamic time warping as preprocessing methods for chromatographic data. J Chemometr..

[7] Gros J, Nabi D, Dimitriou-Christidis P, Rutler R, Arey JS (2012). Robust algorithm for aligning two-dimensional chromatograms. Anal Chem..

[8] Reichenbach SE, Rempe DW, Tao Q, Bressanello D, Liberto E, Bicchi C (2015). Alignment for comprehensive two-dimensional gas chromatography with dual secondary columns and detectors. Anal Chem..

[9] Kim S, Koo I, Fang A, Zhang X (2011). Smith-Waterman peak alignment for comprehensive two-dimensional gas chromatography-mass spectrometry. BMC Bioinf..

[10] Smith TF, Waterman MS (1981). Identification of common molecular subsequences. J Mol Biol..

[11] Robinson MD, De Souza DP, Keen WW, Saunders EC, McConville MJ, Speed TP (2007). A dynamic programming approach for the alignment of signal peaks in multiple gas chromatography-mass spectrometry experiments. BMC Bioinf..

[12] Hoffmann N, Wilhelm M, Doebbe A, Niehaus K, Stoye J (2013). BiPACE 2D—graph-based multiple alignment for comprehensive 2D gas chromatography-mass spectrometry. Bioinf..

[13] Wang B, Fang A, Heim J, Bogdanov B, Pugh S, Libardoni M (2010). DISCO: distance and spectrum correlation optimization alignment for two-dimensional gas chromatography time-of-flight mass spectrometry-based metabolomics. Anal Chem..

[14] Oh C, Huang X, Regnier FE, Buck C, Zhang X (2008). Comprehensive two-dimensional gas chromatography/time-of-flight mass spectrometry peak sorting algorithm. J Chromatogr A..

[15] Kim S, Fang A, Wang B, Jeong J, Zhang X (2011). An optimal peak alignment for comprehensive two-dimensional gas chromatography mass spectrometry using mixture similarity measure. Bioinf. (Oxford, England).

[16] Ladislavová; N. DA_2DCHROM - data alignment. 1.0 ed: Zenodo; 2022. 10.5281/zenodo.7040975

[17] Kim S, Koo I, Jeong J, Wu S, Shi X, Zhang X (2012). Compound identification using partial and semipartial correlations for gas chromatography–mass spectrometry data. Anal Chem..

[18] Ladislavová N, Pojmanová P. DA_2DCHROM - sample dataset. In: Zenodo, editor. 2022. 10.5281/zenodo.7068336

[19] Pojmanová P, Ladislavová N. 2DGCTOF Human Skin scent samples dataset. In: Zenodo, editor. 1.0 ed2022. 10.5281/zenodo.7307846

[20] Pojmanová P, Ladislavová N, Urban Š (2021). Development of a method for the measurement of human scent samples using comprehensive two-dimensional gas chromatography with mass detection. Separations.

[21] Justel A, Peña D, Zamar R (1997). A multivariate Kolmogorov-Smirnov test of goodness of fit. Stat Probabil Lett..

[22] Powers DM. Evaluation: from precision, recall and F-measure to ROC, informedness, markedness and correlation. arXiv preprint arXiv:201016061. 2020. 10.48550/arXiv.2010.16061

[23] Stein SE, Scott DR (1994). Optimization and testing of mass spectral library search algorithms for compound identification. J Am Soc Mass Spectr..

