# Supplementary material for: DA_2DCHROM — a data alignment tool for applications on real GC × GC–TOF samples
Source: Anal Bioanal Chem. 2023 Apr 10;415(13):2641–51. doi: 10.1007/s00216-023-04679-7 (PMC10149467; doi:10.1007/s00216-023-04679-7)
Supplement: Supplementary file 1 — Supplementary file1 (PDF 95 KB) [file 216_2023_4679_MOESM1_ESM.pdf]

Sample collection

First, the volunteers washed their hands with non-perfumed soap (Cormen, CZ), rinsed them under a warm tap, and dried the hands in the air to dryness. Subsequently, the volunteers rubbed their palms to activate the scent glands for 5 minutes. The volunteers received approximately 70 small glass beads (3 mm ⌀). Volunteers were holding and rubbing the beads in both hands for 10 minutes. After the completion of the procedure, the beads were transferred to vials and sealed. The sampling of each volunteer took place once a week for period of 10 weeks.

Sample preparation

2 mL of ethanol (>= 99.8%, Penta CZ) were added to each vial with the collected scent sample. The sample vials were placed in an ultrasonic bath (Fisher Scientific, UK) for 10 minutes and then on a shaker (1000 rpm, IKAVIBRAX VXR basic, Germany) for an additional 10 minutes. The solution was pipetted into 2 mL champagne vials and dried under reduced pressure (concentrator Genevac EZ-2, USA) to complete dryness. Before the analysis, the sample was dissolved in 70 µL of ethanol.

Measurement

The samples were measured on following setup: LECO Pegasus 4D-C detector (LECO Corp, USA), a 7890B gas chromatograph (Agilent, USA), and a MPS robotic MPS multipurpose sampler autosampler from Gerstel (Germany). The first dimension column was a 30 m long Rtx-200 (0.25 mm; 0.25 µm - Restek, USA) with an additional 2 m of the same column as the precolumn. A 1 m Thermo-5HT (0.25 mm; 0.25 µm - Thermo Scientific, USA) was used as a second column. Helium (purity ≥ 99.9995 from Linde CZ) was used as the carrier gas with 1.5 mL / min flow. The temperature program was the following: 40 °C - 2 minutes hold - 5 °C/min ramp - 320 °C - 10 minutes hold. The modulator offset against the secondary oven was set at 15 ° C and the secondary oven offset against the primary oven was set at 5 ° C. There were three modulation periods during the run: 6s (start→1700th s), 8s (1700th to 2588th s), and 10s (2588th→ end). The injection chamber was heated to 280 ° C. The injection volume of the sample was 1µL (splitless). The temperature for the transfer line was set to 280 ° C and for the ion source was se to 250 ° C. The electron ionization energy was set to 70eV. 29-800 m/z fragments were scanned with 200 spectra per second rate.
