# Supplementary material for: DA_2DCHROM — a data alignment tool for applications on real GC × GC–TOF samples
Source: Anal Bioanal Chem. 2023 Apr 10;415(13):2641–51. doi: 10.1007/s00216-023-04679-7 (PMC10149467; doi:10.1007/s00216-023-04679-7)
Supplement: Supplementary file 3 — Supplementary file3 (PDF 16 KB) [file 216_2023_4679_MOESM3_ESM.pdf]

Table of standards - Summary of all chemical compounds which were contained in the standard mixture. All standard samples were measured under the same conditions as samples from system_3. The concentration of each compound was 20 mg/mL.

Chemical Compound	Chemical Compound	Chemical Compound	Chemical Compound
Tridecane	Octadecane	Tricosane	Myristyl myristate
2-Decanone	Tridecanoic acid, ethyl ester	Octadecanoic acid, ethyl ester	Dotriacontane
Tetradecane	Nonadecane	Tetracosane	Tritriacontane
2-Undecanone	2-Pentadecanone	Pentacosane	Octadecanoic acid, dodecyl ester
Pentadecane	Tetradecanoic acid, ethyl ester	Hexacosane	Tetratriacontane
Decanoic acid, ethyl ester	Eicosane	Heptacosane	Pentatriacontane
2-Dodecanone	Pentadecanoic acid, ethyl ester	Octacosane	Hexadecanoic acid, hexadecyl ester
Hexadecane	Heneicosane	Nonacosane	Hexatriacontane
Undecanoic acid, ethyl ester	Hexadecanoic acid, ethyl ester	Tetradecanoic acid, dodecyl ester	Heptatriacontane
Heptadecane	Docosane	Triacontane	Hexadecanoic acid, octadecyl ester
2-Tridecanone	2-Octadecanone	Hentriacontane	Octatriacontane
Dodecanoic acid, ethyl ester	Heptadecanoic acid, ethyl ester	9-Hexadecenoic acid,dodecyl ester, (Z)-	Octadecanoic acid, octadecyl ester
